# Rhizosphere microbiome influence on tomato growth under low-nutrient settings

**DOI:** 10.1093/femsec/fiaf019

**Published:** 2025-02-25

**Authors:** Gerardo Mejia, Angélica Jara-Servin, Cristóbal Hernández-Álvarez, Luis Romero-Chora, Mariana Peimbert, Rocío Cruz-Ortega, Luis D Alcaraz

**Affiliations:** Laboratorio de Genómica Ambiental, Departamento de Biología Celular, Facultad de Ciencias, Universidad Nacional Autónoma de México, 04510 Mexico City, Mexico; Posgrado en Ciencias Bioquímicas, Universidad Nacional Autónoma de México, 04510 Mexico City, Mexico; Laboratorio de Genómica Ambiental, Departamento de Biología Celular, Facultad de Ciencias, Universidad Nacional Autónoma de México, 04510 Mexico City, Mexico; Laboratorio de Genómica Ambiental, Departamento de Biología Celular, Facultad de Ciencias, Universidad Nacional Autónoma de México, 04510 Mexico City, Mexico; Laboratorio de Genómica Ambiental, Departamento de Biología Celular, Facultad de Ciencias, Universidad Nacional Autónoma de México, 04510 Mexico City, Mexico; Departamento de Ciencias Naturales, Unidad Cuajimalpa, Universidad Autónoma Metropolitana, 05348 Mexico City, Mexico; Laboratorio de Alelopatía, Departamento de Ecología Funcional, Instituto de Ecología, Universidad Nacional Autónoma de México, 04510 Mexico City, Mexico; Laboratorio de Genómica Ambiental, Departamento de Biología Celular, Facultad de Ciencias, Universidad Nacional Autónoma de México, 04510 Mexico City, Mexico

**Keywords:** 16S rRNA gene sequencing, fertilizer reduction, low-nutrient conditions, root core microbiome, shotgun metagenomics, tomato (*Solanum lycopersicum* L.) hydroponics

## Abstract

Studies have suggested that reduced nutrient availability enhances microbial diversity around plant roots, positively impacting plant productivity. However, the specific contributions of rhizosphere microbiomes in nutrient-poor environments still need to be better understood. This study investigates tomato (*Solanum lycopersicum* L.) root microbiome under low-nutrient conditions. Plants were grown in hydroponics with soil-derived microbial community inoculations. We hypothesized that nutrient limitation would increase the selection of beneficial bacterial communities, compensating for nutrient deficiencies. We identified 12 294 operational taxonomic units across treatments and controls using 16S rRNA gene sequencing. Increased plant biomass was observed in treatments compared to controls, suggesting a role for the microbiome in mitigating nutrient limitations. The relative abundance of genera such as *Luteolibacter* and *Sphingopyxis* relative abundance correlated with plant phenotypic traits (*P* ≤ .05), and their presence was further validated using shotgun metagenomics. We annotated 722 677 protein families and calculated a core set of 48 116 protein families shared across all treatments and assigned them into bacteria (93.7%) and eukaryota (6.2%). Within the core bacterial metagenome, we identified protein families associated with pathways involved in positive plant interactions like the nitrogen fixation. Limited nutrient availability enhanced plant productivity under controlled conditions, offering a path to reduce fertilizer use in agriculture.

## Teaser

Plants with nutrient half of their suggested nutrients increased their bacterial diversity. Some bacteria communities enabled the plant to overcome the growth limitations. Promising leads to sustainability.

## Introduction

Tomato (*Solanum lycopersicum* L.), domesticated in Mexico and Peru, is a widely consumed staple with high nutritional value, and its cultivation area has doubled over the past two decades (Jaiswal et al. 2020, Tieman et al. 2017). Beyond its agricultural importance, the tomato serves as a model organism in scientific research due to its sequenced genome, diverse genetic tools, short life cycle, and role in studying plant–microbe interactions (Adedayo et al. 2022, van Rengs et al. 2022). A key concept in plant–bacteria interactions is the two-step model for microbiome acquisition, which describes the mutual selection between plants and soil microorganisms, shaping the rhizosphere and endosphere microbiomes (Bulgarelli et al. 2013, Sasse et al. 2018). This model has been validated in tomatoes, revealing a core microbiome influenced by diverse soil geochemistries (Barajas et al. 2020). Additionally, the interaction between the tomato microbiome and host selection is reciprocal, as specific bacterial genera, such as *Streptomyces, Bacillus, Pseudomonas*, and *Flavobacterium*, associate with tomato quantitative trait loci (QTLs) and participate in essential metabolic functions like iron and sulfur metabolism and vitamin synthesis (Oyserman et al. 2022).

Agricultural practices, including chemical fertilization, can deplete plant-available nutrients and reduce belowground diversity, leading to land degradation and environmental pollution (Pahalvi et al. 2021). Additionally, overapplication of nitrogen fertilizers can cause soil acidification, increasing toxic aluminum levels that inhibit root growth (Kopittke et al. 2019). An alternative approach to reducing fertilizer use involves studying plant microbiomes under low-nutrient availability. Microbes play crucial roles in nitrogen fixation, phosphorus mobilization, and overall plant health, as shown in nutrient-stressed soybean and maize studies (van der Heijden et al. 2008, Meier et al. 2022, Wang et al. 2024). Furthermore, plants in nutrient-poor conditions rely on soil microbes for essential nutrients as a compensatory mechanism for nutrient acquisition. One plant response to low-nutrient availability is the change in root exudate composition, leading to concurrent changes in root-associated microbial communities (Zhu et al. 2016).

Low-nutrient availability has also been associated with high microbial diversity in plant-associated communities (van der Heijden et al. 2008). For example, in rice root microbial community, the application of fertilizers reduced diversity (Sinong et al. 2021). Another study using *Brassica oleracea* and testing the response of the bacterial community to fertilizer type, dosage, plant age, and herbivory showed no significant effect of fertilizer additions on α-diversity (O’Brien et al. 2018). The relationship between microbial diversity and plant productivity, particularly in nutrient-limited conditions, requires further investigation. Understanding microbiome dynamics under low-nutrient availability could provide sustainable solutions to reduce fertilizer use while maintaining soil health and productivity.

Methods for cultivating tomatoes significantly influence the structure of their microbiome. Specific differences in microbial communities have been observed between tomatoes from distinct cultivars grown in soil and those grown hydroponically (Escobar et al. 2021). Soilless and hydroponic cultivation methods offer an alternative approach, as they reduce the risk of pathogen infestation and prevent the accumulation of soil-borne pathogens over time (Anzalone et al. 2022). In hydroponic systems, most microorganisms originate from plants, seeds, water, insects, and personnel. However, microbial communities in hydroponic conditions exhibit lower variabiliy compared to those in soil microbiomes (Escobar et al. 2021, Anzalone et al. 2022).

We hypothesize that low-nutrient environments increase selection pressure for root-associated bacteria that compensates for nutrient scarcity. This study aims to identify and characterize bacterial root communities and their genetic traits in *S. lycopersicum* under nutrient-limited conditions providing insights into their adaptation and contribution to plant health. We used a hydroponic system to control nutrient availability and cultivate plants under nutrient-limited conditions. Additionally, we performed a comparison of bacterial communities and their associated functions between tomato plants grown in hydroponics and those grown in soil. We also calculated the core microbiome and metagenome of tomato under both conditions, resulting in the identification of bacteria and predicted proteins highly associated with tomato, which could contribute to better understanding the mechanisms behind tomato adaptation to different environmental conditions.

## Materials and methods

### Soil sampling used as inoculants

We collected seven distinct soil samples and used them as inocula to describe the bacterial communities that could potentially colonize tomato roots under low-nutrient conditions. To investigate different bacterial communities present across different soil types, sampling locations were selected to represent diverse vegetation types, including forests, grasslands, and agricultural areas. Each 2 kg sample was stored in sterile bags and refrigerated at 4°C until inoculation experiments began. Metadata for each site included the collection date, geographic coordinates, altitude, and observations documented (Table S1).

### Plant growth conditions

We regulated nutrient concentration and mechanical soil properties using a hydroponic system with plastic cups and nylon netting containers (Alatorre-Cobos et al. 2014). The cups were sterilized with 70% ethanol and 4.5% commercial sodium hypochlorite. *Solanum lycopersicum* L. cv. Rio Grande seeds were surface-sterilized with 70% ethanol for 1 min, followed by a 2.5% NaClO wash for 2 min, and rinsed with sterile distilled water. The seeds were germinated in chambers for 72 h at 21°C with a 12-h light/dark cycle before being transferred to hydroponic containers, with five plants per container.

In order to investigate the impact of bacterial communities on plant productivity under low and high nutrient availability conditions, we established treatments with the same soil inoculum in low (t50, 50% of the recommended hydroponic fertilizer) and high nutrient availability (t100, 100% of the recommended hydroponic fertilizer). Each treatment included 5 g of soil added to the hydroponic medium, along with three pots, each containing four plants. We established controls containing only fertilizer (f100 and f50) and Nutritional Controls (NC) with sterilized inoculum for both conditions (NC50 and NC100). The sterilized inocula allowed the evaluation of nutritional contributions without the native microbiota NC. The plants were grown for 30 days in a greenhouse under natural light and dark conditions. Phenotypic traits were measured at the end of the experiment.

**Figure 1.**
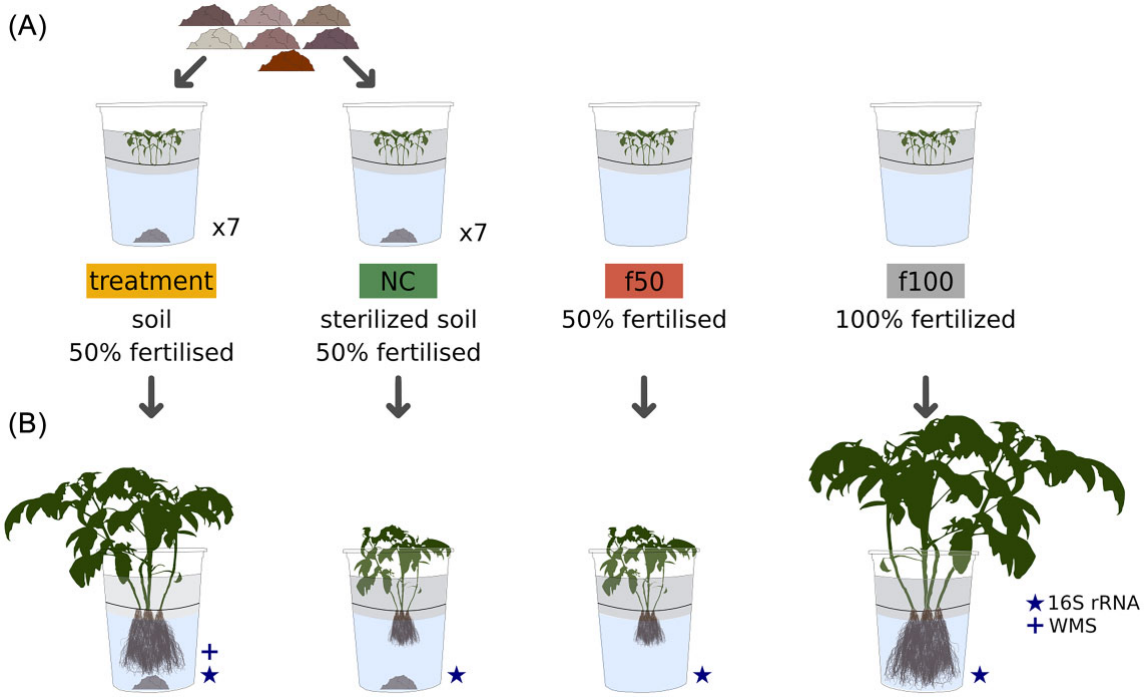
Experimental layout. The diagram summarizes the methods to evaluate the effect of rhizosphere communities and fertilization levels in tomato growing in a hydroponic system: (A) treatment groups: nonsterilized soil with microbial inoculants and 50% fertilization; seven different soils were tested individually. Nutrient Control, one for each soil tested (NC): sterilized soil with 50% fertilization. 50% Fertilizer Control (f50): reduced nutrient conditions without microbial inoculation. 100% Fertilizer Control (f100): Full nutrient conditions without microbial inoculation. (B) We selected inoculated plants grown in nonsterilized soil, which showed better growth under 50% fertilization compared to those grown in sterilized soil and f50, highlighting the effects of microbial communities on plant biomass production. WMS: whole metagenome sequencing. Reproduced from Mejia et al. (2024) under CC-BY license.

To describe bacterial communities under low-nutrient availability, we applied treatments with 50% of the recommended fertilizer. Seven different soil samples were used as separate microbial inocula in the hydroponic system across treatments (Fig. 1). Each treatment included 5 g of soil added to the hydroponic medium, along with three pots, each containing five plants. We incorporated NC controls 50% fertilized (Fig. 1). To evaluate growth under varied nutrient conditions we included two different controls: one with 100% fertilization (f100) and another with 50% fertilization (f50), both without soil (Fig. 1). The plants were grown for 60 days in a greenhouse under natural light and dark conditions.

The experiment used 240 plants across treatments and controls. We evaluated the seven collected soils, testing 15 plants per soil type. Phenotypic traits were measured at the end of the experiment. We compared plant phenotypic traits—stem length, dry weight, stem diameter, and leaf area—between treatments and controls to identify significant growth improvements. Stem length was measured from the base to the apical meristem (Ohta and Ikeda 2015). Stem diameter was measured below the cotyledons with an electronic Vernier (Jwa and Walling 2001). Leaf area was estimated using aerial photographs analysed with GIMP (GIMP Development Team 2021) and ImageJ (Schneider et al. 2012). Plants were oven-dried at 45°C for 10 days to determine dry weight. Based on plant phenotypes, we selected three inocula (soils) with positive effects on plant biomass production for further analysis of the bacterial communities in the respective roots using whole metagenome shotgun and 16S amplicon sequencing.

The hydroponic medium used for the 100% fertilized pots (f100 and t100) contained per liter: 0.5 g MgSO_4_, 0.45 g KNO_3_, 0.22 g KH_2_PO_4_, 0.8 g citric acid, 0.1 g EDDHA–Fe, 1 g Ca(NO_3_)_2_, and 0.3 g STEM (S 13%, B 1.35%, Cu 2.3%, Fe 7.5%, Mn 8%, Mo 0.04%, and Zn 4.5%). Treatments (t50), f50, and NC used a medium with half of these nutrient concentrations (Table S2). NC were established by autoclaving the inocula twice (15 min at 121°C, 15 psi, with a 24-h interval).

### Metagenomic DNA processing, sequencing, and analyses

Rhizosphere and endosphere fractions of our hydroponic plants were obtained following Lundberg et al. (2012). DNA was extracted from the rhizosphere and endosphere using the PowerSoil DNA Extraction Kit (Qiagen, Hilden, Germany). Rhizosphere and endosphere fractions were analysed separately for 16S rRNA gene sequencing and mixed for shotgun metagenomic sequencing. For 16S rRNA gene sequencing, the V3–V4 regions were amplified (Herlemann et al. 2011) and sequenced on an Illumina MiSeq platform. The 16S rRNA gene was amplified using 341F/805R primers targeting the V3–V4 regions, following the Illumina MiSeq protocol with 5′ overhangs. Duplicate polymerase chain reaction (PCR) for each sample were performed with Pfx platinum polymerase under the following conditions: 95°C for 3 min; 5 cycles at 94°C for 30 s, 55°C for 30 s, and 68°C for 30 s; then 25 cycles at 94°C for 5 s and 68°C for 30 s; and a final extension at 68°C for 5 min. Products were purified with the SV Wizard PCR Purification Kit (Promega, WI, USA). Shotgun metagenomic sequencing of both fractions was conducted using the Illumina NextSeq 2 × 150 at the LANGEBIO Genomic Services Laboratory.

Detailed bioinformatics and statistical analysis procedures for 16S sequencing are available on GitHub (genomica-fciencias-unam/tomato-hydroponics). Briefly, raw reads were trimmed using the FASTX-Toolkit (“fastx_trimmer”) (Hannon 2010) when the Phred quality score was below 20. Forward reads were trimmed to 250 bases, and reverse reads to 220 bases. The reads were assembled with PANDAseq (Masella et al. 2012) using a minimum overlap of 250 bp and a quality threshold 0.6. Operational taxonomic units (OTUs) were defined at 97% identity and clustered using cd-hit-est v4.7 (Li and Godzik 2006). Singleton sequences, chimaeras (detected by blast_fragments method; Caporaso et al. 2010), mitochondria, and chloroplast sequences were removed. Taxonomy was assigned using BLAST (Altschul et al. 1990) with the SILVA v138.1 database (Quast et al. 2013).

Metagenomic reads were quality-filtered with Trimmomatic v0.36 (Bolger et al. 2014). Sequences matching the *S. lycopersicum* genome (NCBI BioProject: PRJNA66163) were removed with Bowtie2 v2.3.4.1 (Langmead and Salzberg 2012). Quality-filtered sequences were assembled with metaSPADES v3.12.0 (Nurk et al. 2017). Unmapped reads were reassembled with Velvet v1.2.10 using a k-mer size 31 (Zerbino and Birney 2008). Contigs from both assemblies were used to predict open reading frames (ORFs) with Prodigal v2.6.3 (Hyatt et al. 2010). Predicted genes were annotated against the M5nr database (Wilke et al. 2012) using Diamond v0.9.18.119 (Buchfink et al. 2015). Taxonomic assignment of predicted proteins was performed with Kraken2 v2.1.2 (Wood et al. 2019) using default parameters and the PlusPF database with RefSeq indexes (O’Leary et al. 2016). An abundance table was generated by mapping reads against predicted proteins using Bowtie2. Unannotated proteins were clustered with cd-hit v4.7 (Li and Godzik 2006) at 70% identity.

To compare the predicted protein diversity associated with tomato in hydroponics (this study) to that associated with tomato grown in soil, we retrieved metagenome data from a previous study on soil-grown tomatoes of the same genotype (*S. lycopersicum* var. Río Grande) were retrieved from NCBI BioProject: PRJNA603603 (Barajas et al. 2020) and processed alongside the current sequencing data. For comparative analysis, both datasets were compared using subsamples of each metagenome by selecting 1 × 106 random reads in three replicates.

### Taxonomic identification of bacteria associated with low-nutrient availability and metagenomic analysis

We analysed bacterial taxa in treatments and control groups using the 16S rRNA gene. We calculated relative abundances and employed Pearson’s correlation to examine relationships with plant phenotypic variables, focusing on taxa consistently present with high correlation coefficients (*r*² > 0.7). Additionally, to identify genera potentially involved in compensating for nutrient scarcity, we conducted a differential abundance analysis using the corncob R package (Martin et al. 2020). Genera overrepresented in treatments with low-nutrient availability were identified using DESeq2 v1.10.1 (Love et al. 2014). To identify those genera recruited by tomato in low-nutrient conditions, we calculated genera shared and unique among controls and treatments using a Venn Diagram.

### Comparison of the tomato root microbiome in hydroponic and soil-grown systems, and core microbiome

To understand the role of the cultivation medium in shaping the tomato bacterial communities and the microbiome, we used data generated by Barajas et al. (2020), which includes 16S rRNA amplicons and shotgun sequencing data from tomato roots grown in soil. We compared these data with those generated in the present work, where tomatoes of the same genotype were grown in hydroponics. Hydroponic inocula originated from agricultural soils, differing from soils used for soil-grown tomatoes. Soil-grown tomato data were reanalysed to identify Tomato Core Microbiome (TCM), focusing on the intersection of genera across all samples from both treatments in hydroponic and soil-grown plants.

We defined a Hydroponic Tomato Core Metagenome (HTCMe) from protein families shared across all hydroponic treatments. At the same time, the Tomato Core Metagenome (TCMe) was established from protein families in all root samples from both conditions (hydroponics and soil). Nonmetric dimensional scaling (NMDS) and was used to compare protein families across both datasets.

### Hydroponic bacteria pangenome analysis

The 16S amplicon analysis identified genera with high correlation values to plant traits, overrepresented in treatments and present in the core microbiome. We searched for their pangenomes in the hydroponic rhizosphere metagenomes. Using Prokka v1.12, we annotated reference genomes from selected bacterial species: *Flavobacterium* (43 genomes), *Hyphomicrobium* (6), *Luteolibacter* (8), *Methyloversatilis* (3), and *Sphingopyxis* (19), chosen for their association with tomatoes and prevalence in our samples. Roary software calculated pangenomes aligned with hydroponic metagenomic reads using Promer v3.23. Alignment percentage identity and coverage were visualized with identity graphs created using the promer_deid_v9gmv.py script (available on GitHub).

### Data analyses

Statistical analyses of plant phenotypes were conducted using R v3.5.1 (R Development Core Team 2003), with ggplot2 v3.3.0 (Wickham 2016) for plot design. Diversity analysis utilized R’s phyloseq v1.24.2 (McMurdie and Holmes 2013). Spearman correlations between genera abundance and plant phenotype were analysed using corrplot v0.84 (Wei 2021), *P*-values were adjusted for multiple comparisons using the Benjamini–Hochberg method. Genera analysis in hydroponics and core microbiome calculations employed the Venn diagram v1.6.20 (Chen 2022). Protein core metagenome was determined with UpSet v1.4.0 (Conway et al. 2017). Differential taxa and predicted proteins were calculated using DESeq2 v1.10.1 (Love et al. 2014) in R v3.2.2. constrained analysis of principal coordinates (CAP) analysis used the Bray–Curtis dissimilarity score, constrained by plant phenotypic traits. NMDS ordination used Bray–Curtis dissimilarity scores between predicted proteins of tomato hydroponic and soil systems.

## Results

### Plant productivity and microbiome diversity under low-nutrient availability

We hypothesize that nutrient limitation drives the selection of bacterial communities that compensate for nutrient deficiencies. To test this, we used *S. lycopersicum* plants in a hydroponic system to investigate the effect of microorganisms on plant productivity under low-nutrient availability. We implemented treatments with soil added to the hydroponic medium as a microbial inoculum under low-nutrient availability (50% fertilization, t50). We contrasted these conditions with plants growing under high nutrient availability and the same soil inoculum (100% fertilization, t100). In this experiment, we included controls containing only fertilizer (f100 and f50) and controls with the sterilized inoculum under low (50% fertilization, NC50) and high (100% fertilization, NC100) nutrient availability. These controls allowed us to evaluate the nutritional contribution of the inoculum as well as the contribution of the fertilizer to plant growth under both conditions.

The presence of the inoculum under high nutrient availability (t100) diminished biomass production compared to f100 control (*t*-test, *P* ≤ .001) (Fig. S1A). Likewise, no significant differences were detected in plant length between t100 and its respective controls NC100 and f100 (Fig. S1B). In contrast, under limited nutrient availability, t50 exhibited higher biomass production and plant length compared to f50 and NC50 (*t*-test, *P* ≤ .001) (Fig. S1A and B and Table S3). These results indicate that bacteria and other microorganisms from the inoculum have a positive impact on plant productivity under low-nutrient availability conditions.

Based on the positive effect of microbial communities on plant productivity under low-nutrient availability, we evaluated multiple inocula and characterize the bacterial community associated with tomato under these conditions. Treatments involved plants with a low-nutrient profile (50% fertilization) and soil as a microbial inoculum. Controls included a group without inoculum with 50% fertilizer (f50) and another with 100% fertilizer (f100) to evaluate growth under different nutrient conditions. Nutrient controls (NC) were prepared with sterilized inoculum and received 50% fertilization (Fig. 1). Our assessment of several plant traits revealed the effects of bacterial communities originating from the inoculum.

The highest average biomass was recorded in the maximum fertilization control (f100 = 4.86 g ± 0.71), followed by the 50% fertilized pots with soil as microbial inoculants (treatment = 2.77 g ± 0.46). Sterilized soil with 50% nutrients produced lower biomass (NC = 1.87 g ± 0.57), while the minimum fertilization control (f50) produced the least biomass (0.83 g ± 0.45).These findings underscore the potential of bacteria and other microorganisms in enhancing plant growth, particularly under nutrient-limited conditions

**Figure 2.**
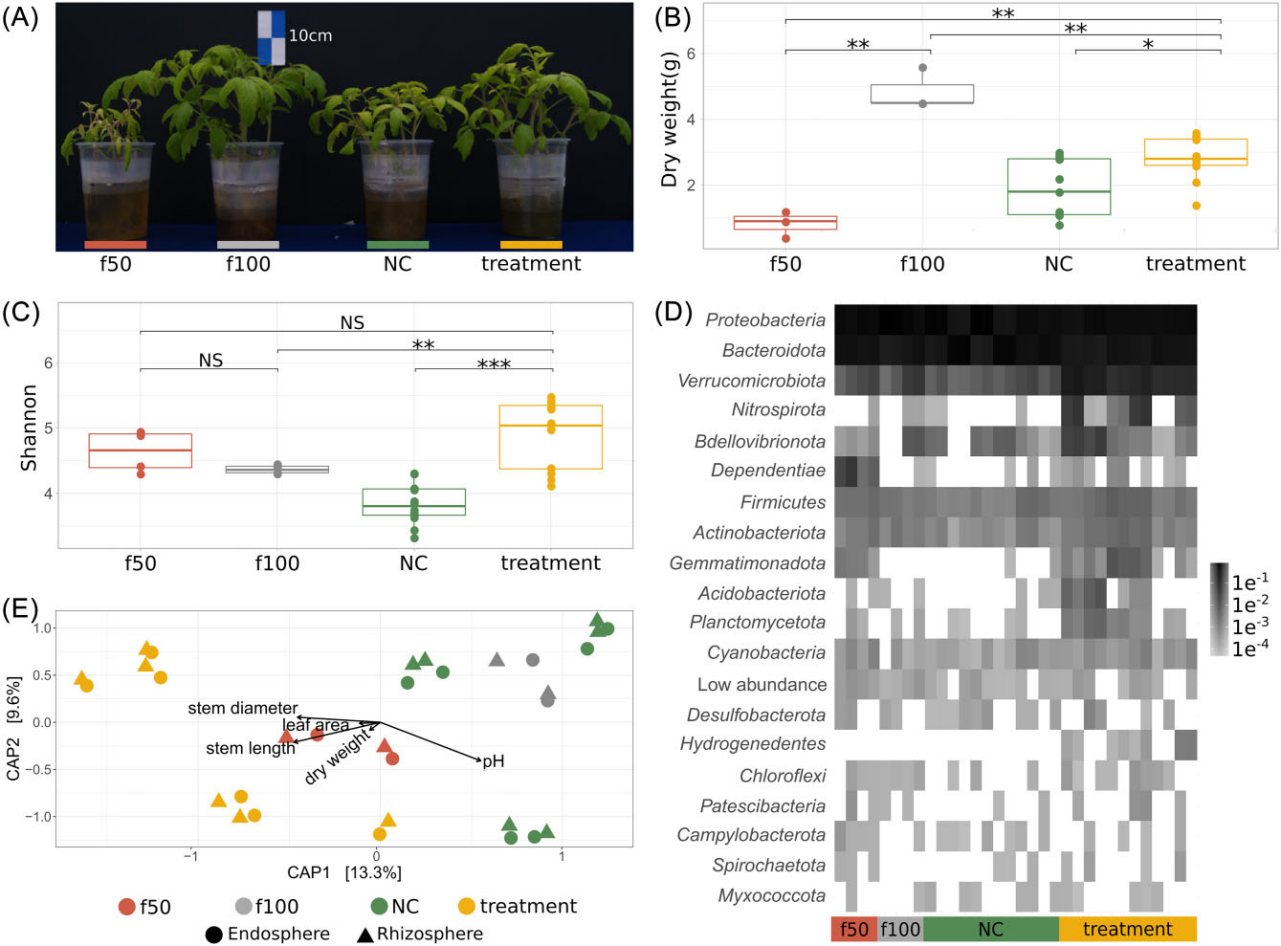
Effect of low-nutrient availability on root-associated bacterial diversity and plant growth. (A) Representative plants from the low-nutrient availability experiment: 50% fertilizer control (red, f50), 100% fertilizer control (gray, f100), NC (green), and treatment (yellow), illustrating visual differences in plant growth. (B) Box plots of plant biomass production, highlighting growth differences under varying nutrient and microbial conditions (*P < .05,**P < .01, and ***P < .001). (C) Box plots showing the Shannon diversity index across treatment groups, indicating variations in microbial diversity. (D) Heatmap depicting the relative abundance of phyla within each treatment and control group, with phyla of low relative abundance (≤ 0.001) shown in the “Low abundance” category. (E) CAP ordination plots based on Bray–Curtis distances, correlating OTUs with plant phenotypic traits and pH across all groups, illustrating the influence of microbial diversity on plant characteristics.

Biomass production in treatments significantly exceeded that of the f50 and NC groups, demonstrating enhanced performance in treatments with inoculum and low-nutrient availability (*t*-test, *P* ≤ .001 and *P* ≤ .05, respectively; Fig. 2A and B). Similarly, the highest stem diameters were observed in f100 (5.60 mm ± 0.29) and treatments (4.81 mm ± 0.16), with treatments showing significant improvements over NC (3.70 mm ± 0.39) and f50 (3.12 mm ± 0.72) (*t*-test, *P* ≤ .001 and *P* ≤ .5, respectively). The stem length of treatments (32.84 cm ± 2.34) was comparable to f100 (37.16 cm ± 4.64), showing no significant difference, despite being significantly longer than those of f50 (20.7 cm ± 4.15) and NC (26.99 cm ± 2.85) (*t*-test, *P* ≤ .05 and *P* ≤ .01, respectively).

No significant differences were observed in leaf area between treatments (1390.79 cm² ± 211.21) and NC (1072.62 cm² ± 336.20) or between treatments and f50 (690.93 cm² ± 451.71). Additionally, wet weights were similar across treatments (10.59 g ± 1.47), f50 (5.86 g ± 3.41), and NC (9.24 g ± 2.18).

The substantial growth observed in treatments, compared to f50 and NC, suggests a beneficial impact of bacterial communities (Fig. 2A and Table S4). In contrast, chlorosis, reduced biomass production, and poor growth performance in the f50 group indicate nutritional stress and suboptimal growth conditions (Fig. 2A).

### Plant root bacteria diversity under low-nutrient availability

We sequenced 3 591 762 reads (average = 284 bp) across 32 samples. After quality control and sequence clustering, we identified 12 294 OTUs (97% 16S rRNA gene identity). These OTUs were classified within the hydroponic dataset, encompassing treatments, NC, and fertilized controls (f100 and f50).

Treatments exhibited the highest richness with 7247 OTUs (Chao1 = 3223.19 ± 321.24), followed by NC with 5265 OTUs (Chao1 = 2812.76 ± 268.88), f50 with 3077 OTUs (Chao1 = 3019.89 ± 666.25), and f100 with 2957 OTUs (Chao1 = 2812.95 ± 323.14). The Shannon index revealed that treatments had the highest bacterial diversity (median = 5.03 ± 0.51), followed by f50 (4.66 ± 0.32). f100 and NC showed reduced diversity (4.36 ± 0.07; 3.80 ± 0.31, respectively). Significant differences were found in the diversity of treatments compared to all other groups (ANOVA, *P* = 3.48×10^−6^; Tukey-HSD, *P*-adj < .05), indicating a robust microbial enrichment under treatment conditions (Fig. 2B).

### Bacterial taxa associated with tomato roots under low-nutrient conditions

Taxonomic assignments of OTUs revealed that Proteobacteria was the dominant phylum across all conditions. However, treatments displayed a lower proportion of Proteobacteria (x̅ = 49.66%) compared to f100 (66.71%), f50 (55.35%), and NC (52.05%). Bacteroidota followed in abundance, with treatments showing a lower percentage (33.09%) compared to f50 (40.89%) and NC (46.54%), while f100 had the lowest (30.16%). Verrucomicrobiota was notably more abundant in treatments (13.59%) than all controls, indicating distinct microbial community structuring under treatment conditions (Fig. 2D).

The 12 294 OTUs were assigned to 818 bacterial genera. *Flavobacterium* was the most abundant in treatments (x̅ = 22.79%) and had a substantial relative abundance growth in NC (45.14%). In contrast, the relative abundance of *Flavobacterium* was lower in f100 (18.65%) and f50 (16.33%). On the other hand, *Allorhizobium*–*Neorhizobium*–*Pararhizobium*–*Rhizobium* (*ANPR*), considered a single genus by the SILVA database, was the most prevalent genus in f100 (29.70%) and f50 (19.13%). However, *ANPR* relative abundance decreased in both treatments (6.49%) and NC (14.66%). Notably, *Luteolibacter* showed a remarkable increase in treatments (11.76%) when compared with NC (0.09%), f100 (0.02%), and f50 (0.009%) (Fig. S2, Table S6, and S7).

**Figure 3.**
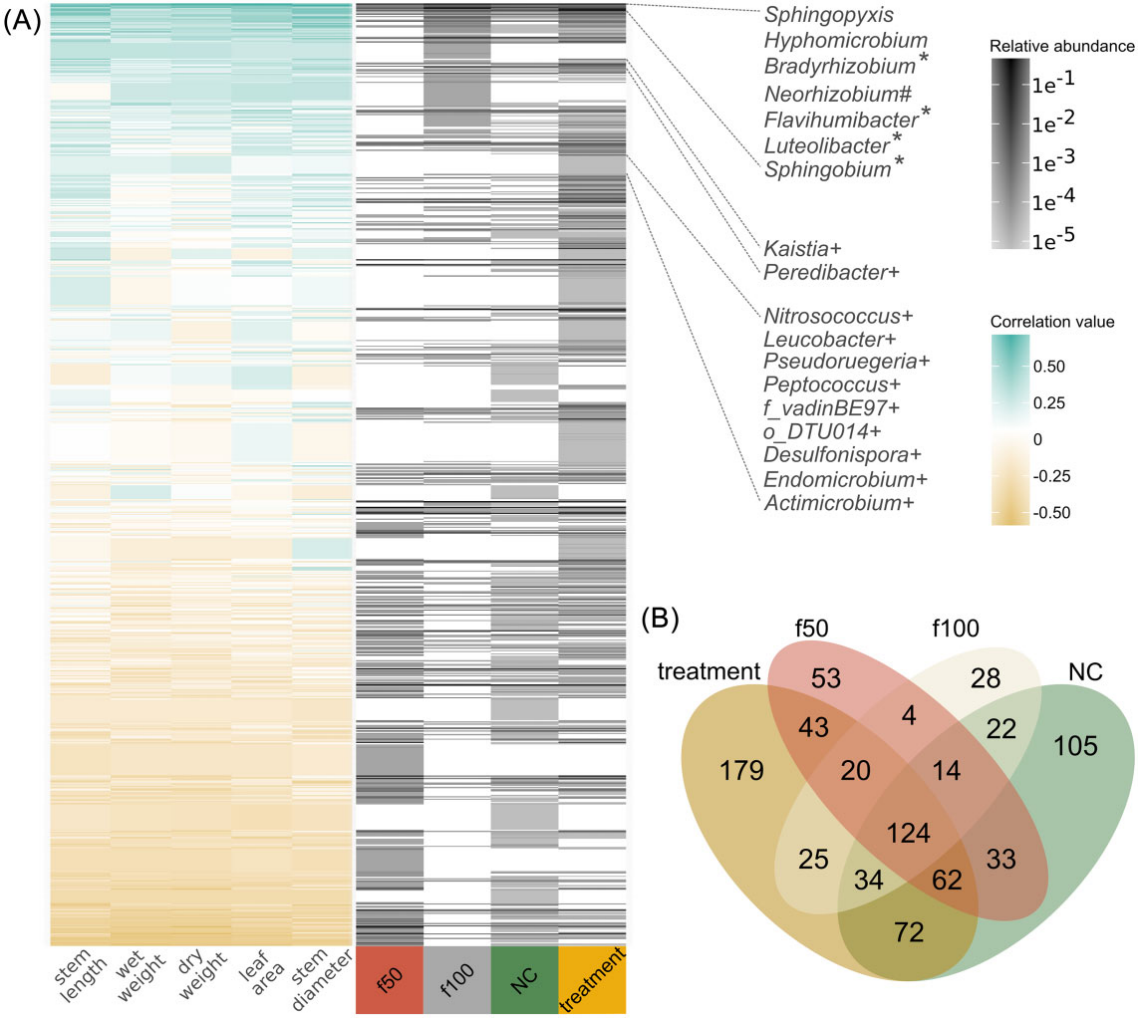
Relationship between microbial genera and plant phenotype. (A) Heatmaps showing the correlation between bacterial genera relative abundance (gray scale) and plant phenotypic traits across treatment and control groups. Genera are sorted by decreasing correlation values, indicating their association with plant growth characteristics. Symbols denote genera overrepresented in treatments (*), and those unique to treatments (+). (B) Venn diagram illustrating the distribution of microbial genera in controls and treatments, highlighting shared and unique communities to emphasize differences in microbial recruitment and establishment under varying conditions.

A CAP was performed on the 16S communities using Bray–Curtis distances (Fig. 2E), explaining 22.9% of the variance in microbial communities, with CAP1 accounting for 13.3% and CAP2 for 9.6%. The treatment groups formed distinct clusters, separate from the controls. Significant differences were found between the treatments, NC, and controls (*P* = 1×10^−4^; Adonis), with controls (f50 and f100) showing greater dispersion. A notable difference was observed between NC (sterilized soils with 50% fertilizer) and treatments (soil inoculum with 50% fertilizer), indicating differences in the bacterial communities. Plant phenotypic traits such as dry weight, stem diameter, stem length, and leaf area were positively related to bacterial communities and its structure in the treatments, based on the CAP analysis (Fig. 2E). Phenotypic traits and bacterial genera abundance suggested key bacterial taxa in plant interaction. Significant Spearman’s correlations (*P* ≤ .05), showed that genera strongly correlated with beneficial plant phenotypes were prevalent in treatments. In contrast, those with low or negative correlations were found in NC and f50 controls (Fig. 3A). Genera such as *Sphingopyxis, Hyphomicrobium, Bradyrhizobium, Neorhizobium, Flavihumibacter, Luteolibacter*, and *Sphingobium* showed the highest correlation with positive plant traits (Table S7). Additionally, differential abundance analysis using corncob R package (Martin et al. 2020) showed a positive and significant association between genera such as *Hyphomicrobium, Luteolibacter*, and *Sphingopyxis* and plant phenotypic traits (Fig. S3). Suggesting they could positively impact plant productivity.

Qualitative and quantitative analyses delineated shared and unique bacterial genera between treatments and controls. A Venn diagram revealed 179 unique genera within treatment groups, suggesting a rich diversity of potentially beneficial microbes (Fig. 3B and Table S8). We found unique genera in treatments, including *Kaistia, Peredibacter, Nitrosococcus, Leucobacter, Pseudoruegeria*, and *Desulfonispora*. Additionally, these genera were positively correlated with plant phenotypes (Fig. 3A).

Sixty-six genera showed significant variations in abundance between treatments. (*P*-adj *≤ 0.05, DESeq2*), including *Caedibacter, Luteolibacter, Flavihumibacter, Sphingobium*, and *Bradyrhizobium*, associated with positive plant traits (Table S9). Additionally, 13 genera showed significant changes in controls, such as *Brevundimonas*, the combined group *ANPR, Phenylobacterium*, and *Flavobacterium*, also positively correlated with plant phenotypes (Table S7). This analysis highlights the dynamic interactions between bacterial communities and plant phenotypic traits under low-nutrient conditions with an inoculum, suggesting bacterial taxa potentially associated with plant growth.

### Shared core taxa of tomato microbiome under hydroponics and soil growth

**Figure 4.**
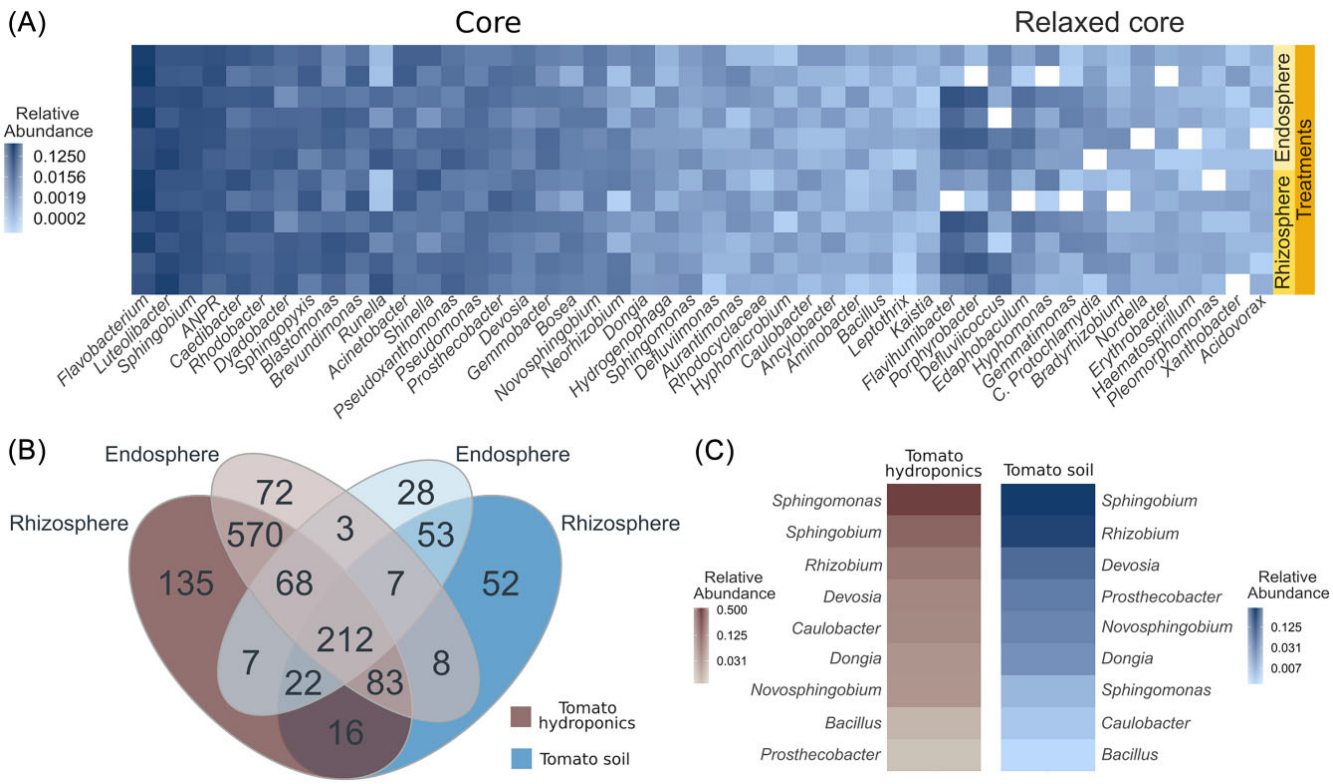
Analysis of the TCM in hydroponic and comparison with soil systems. (A) Heatmap showing the relative abundances of genera within the HTCM. Relaxed core was determined by accounting for the presence of each genus in all samples except one. *ANPR*. (B) The Venn diagram shows the shared genera between tomatoes in soil and hydroponics. (C) Relative abundance of genera found in a strict core for hydroponic and soil rhizosphere samples.

Our research identified a core microbiome in hydroponic tomato roots, consistent across all treatments and comprising 34 genera, collectively known as the Hydroponic Tomato Core Microbiome (HTCM; Fig. 4A). HTCM include *Flavobacterium, Luteolibacter, Sphingobium, ANPR, Caedibacter, Rhodobacter, Dyadobacter*, and *Sphingopyxis*. We also identified a relaxed core featuring genera present in at least 11 of the 12 treatment samples, such as *Flavihumibacter, Porphyrobacter, Defluviicoccus, Edaphobaculum, Hyphomonas, Gemmatimonas, Candidatus Protochlamydia, Bradyrhizobium, and Nordella* (Fig. 4A).

Comparing hydroponic and soil-grown tomato roots (Barajas et al. 2020) revealed 212 common genera, 133 unique to hydroponics, and 777 exclusive to soil (Fig. 4B and Table S10). The HTCM and Soil Tomato Core Microbiome (STCM) comparison showed nine genera common to both systems, forming the TCM. This hybrid core includes *Sphingomonas, Sphingobium, Rhizobium, Devosia, Caulobacter, Dongia, Novosphingobium, Bacillus*, and *Prosthecobacter* (Fig. 4C).

The relative abundance of these genera varied, with *Sphingobium* more prevalent in hydroponics and *Sphingomonas* more abundant in soil, highlighting their adaptability and crucial role in the tomato root microbiome across different conditions.

### Tomato root metagenomic diversity under low-nutrient availability

Hydroponic treatment metagenomes generated 126 030 926 raw reads. After quality control and filtering out host plant sequences (*S. lycopersicum* L.), 114 475 444 sequences remained, assembling into 5 239 742 contigs with an average length (N50) of 1506 base pairs. Protein prediction identified 5 196 182 proteins grouped into 1 984 648 protein families at 70% identity. Of these, 722 677 proteins were annotated using the M5nr database (Table S11).

Notable proteins in hydroponic treatments included the IS110-like element ISPa71 transposase (0.05% relative abundance) and others such as hypothetical proteins hp_5547 (0.037%) and hp_304 (0.028%), a DUF11 domain-containing protein (0.023%), glycosyltransferase (0.022%), and a Z1 domain-containing protein (0.020%). Proteins involved in heavy metal efflux, like the CusA/CzcA family RND transporter, also showed a 0.020% abundance (Table S13).

NMDS analysis showed distinct clustering of predicted proteins of hydroponic and soil-grown tomato metagenomic proteins (Fig. S4A). Hydroponic samples contained significantly more proteins (334 724) than soil samples (142 737; *P* ≤ .01 Wilcoxon). The Shannon–Weaver diversity index was higher in hydroponic samples (H' = 11.62 ± 0.22) compared to soil samples (H' = 10.97 ± 0.28) (Table S14 and Fig. S5), likely due to higher sequencing coverage in hydroponics (42 × 10^6^ reads versus 29 × 10^6^ in soil).

DESeq2 analysis identified significant differences between protein families in hydroponic (1388 families) and tomato soil metagenomes (330 families) (*P*-adj ≤ .001) (Fig. S4B). Proteins like the IS110-like element ISPa71 transposase and the TonB-dependent receptor were more prevalent in hydroponic samples (Table S15). Cluster of Orthologous Groups (COG) categorization showed abundant categories in hydroponics: replication, recombination, and repair (L COG); unknown functions (S COG); amino acid transport and metabolism (E COG); translation, ribosomal structure, and biogenesis (J COG); and inorganic ion transport and metabolism (P COG) (Fig. S4C).

### Shotgun metagenomics taxa diversity

The total metagenome provides an unbiased perspective on microbial diversity, avoiding PCR amplification biases associated with 16S amplicons, ITS, and other marker genes (de Lilo et al. 2006, Tessler et al. 2017). Taxonomic assignment of all metagenomic shotgun contigs was conducted using the Kraken2 (Wood et al. 2019) program. For the hydroponic-grown tomatoes, most of the sequences were assigned to bacteria (93.72%), followed by eukaryotes (6.2%), archaea (0.008%), and viral sequences (0.025%). Among the eukaryotes, most sequences were identified as human (0.76%) and fungi (0.28%), while the remaining 5.16% comprised a diverse array of unicellular eukaryotes. A complete table of taxonomic assignments and abundances is available in the supplementary materials (Table S12).

We identified a notable mix of potentially phytopathogenic, saprophytic, and biocontrol fungi and fermenting yeasts, basidiomycetes, algae, protozoans, and parasites within the eukaryotic diversity. The detected phytopathogenic genera included *Cercospora, Fulvia, Zymoseptoria, Colletotrichum, Botrytis, Fusarium, Pyricularia, Rhizoctonia, Ustilaginoidea*, and *Puccinia* (Doehlemann et al. 2017). The sampled plants exhibited no symptoms despite known plant pathogens and appeared healthy. We also identified fungi capable of degrading lignocellulose and organic matter, including *Marasmius, Thermothelomyces, Thermothielavioides*, and *Neurospora* (Wang et al. 2023, Caputo et al. 2024). Notably, *Thermothelomyces* has demonstrated the potential for degrading bacterial biofilms, including *Escherichia coli* (Samaniego et al. 2024). Additionally, fungi with antibiotic production capabilities and biocontrol potential, such as *Aspergillus, Penicillium*, and *Talaromyces*, were detected (Nicoletti and Trincone 2016, Boruta et al. 2021, Abbas et al. 2024). Diatoms, like *Thalassiosira pseudonana* and *Haslea ostrearia*, were identified in the system. Fewer than 40 contigs corresponded to various protozoa and zoonotic parasites, including *Plasmodium malariae, Cryptosporidium* spp., and *Theileria* spp. This finding highlights the importance of environmental monitoring from a One Health perspective, particularly for tracking potentially pathogenic organisms in nonhuman environments.

Regarding archaea, we found multiple species of halophilic (e.g. *Halobaculum* and *Halorubrum*), methanogenic (e.g. *Methanothermobacter* and *Methanobacterium*), ammonia-oxidizing archaea (AOA) (e.g. *Nitrosocosmicus*). *Nitrosocosmicus* has already been reported to be associated with plant rhizospheres and plays key roles as AOA archaea (Alves et al. 2019, Lee et al. 2024). Viruses were present in low abundance (0.025%), yet we found virus-infecting algae (e.g. ATCV1 and *Micromonas* viruses), fish-infecting viruses (e.g. Koi herpesvirus), and insect-infecting viruses (e.g. entomopoxvirus) for moths and lepidopterans. Although bacterial sequences dominated the analysed microbiomes, these findings demonstrate that shotgun sequencing enables the detection of broader microbial diversity, including low-abundance taxa. Given our focus, subsequent analyses will center on the bacterial component.

### TCMe analysis

We identified a core set of 48 116 protein families in all hydroponic tomato treatments, termed the HTCMe (Fig. S6A and Table S16). The HTCMe includes various proteins such as the IS110-like element ISPa71 family transposase, DUF11 domain-containing protein, glycosyltransferase, Z1 domain-containing protein, CusA/CzcA family heavy metal efflux RND transporter, and PAS domain S-box protein. Notably, it also contains proteins involved in plant growth promotion, including tRNA dimethylallyl transferase MiaA, indole acetamide hydrolase, tryptophan decarboxylase, aldehyde dehydrogenase, and components of the nitrogenase enzyme complex (*nifD, nifK, nifH, nifA, nifB, nifE, nifN, nifW*, and *nifZ*).

Using metagenomic data from soil-grown tomatoes (Barajas et al. 2020), we established the Soil-grown Tomato Core Metagenome (STCMe) (Fig. S6B and Table S17). We also defined the TCMe as protein families common to all soil-grown and hydroponic tomato rhizosphere samples. The TCMe comprises 663 protein families, including BamA/TamA family outer membrane proteins, TamB domain-containing proteins, patatin-like proteins, aspartate aminotransferase family proteins, TonB-dependent receptors, NAD-glutamate dehydrogenase, and glutamate synthase large subunit. These proteins were consistently detected across all samples, underscoring their fundamental role in tomato physiology across different growing conditions (Fig. S6C and Table S18).

### Key bacteria identification in metagenomes

**Figure 5.**
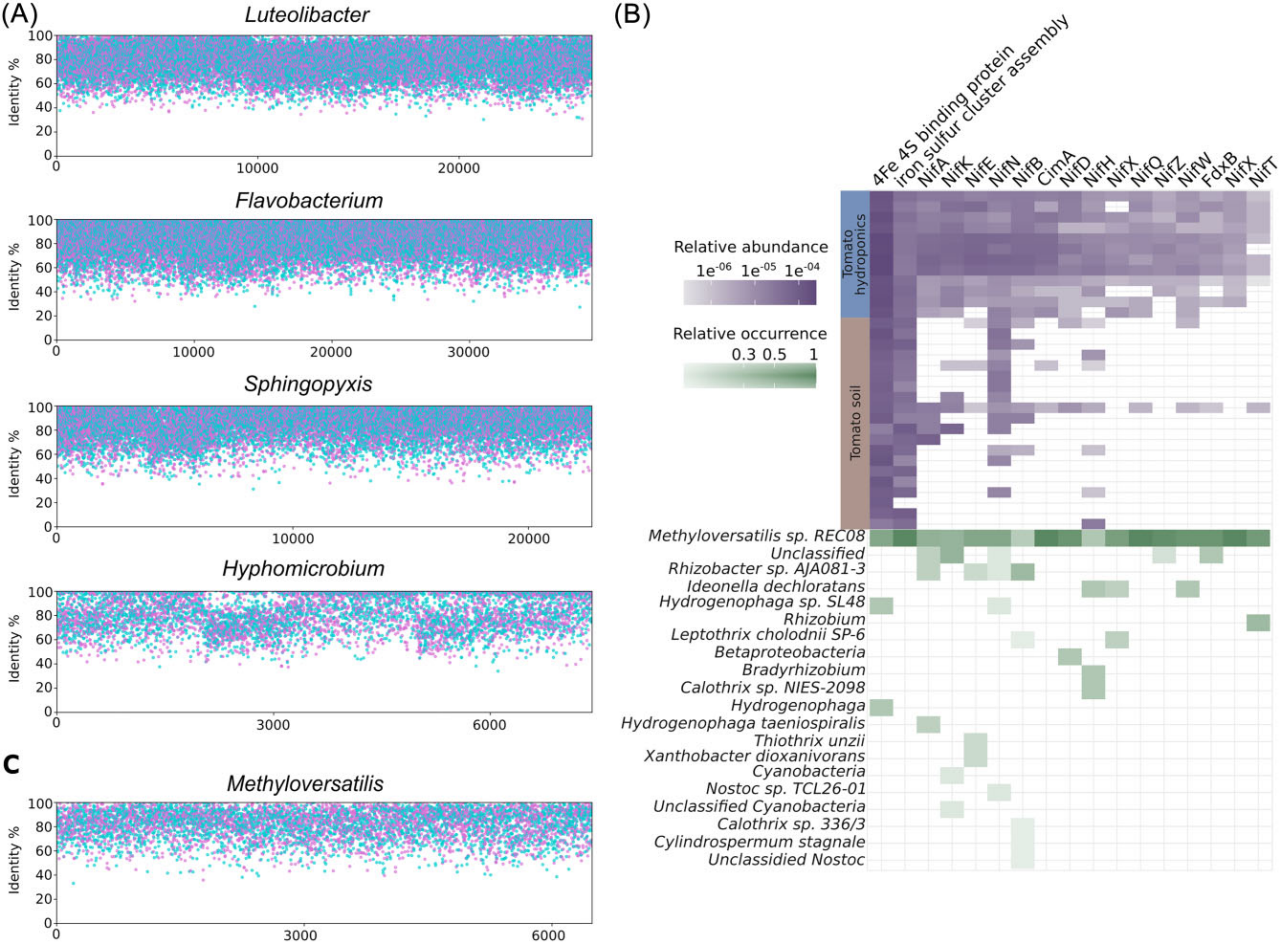
Pangenomic analysis of key microbial genera in tomato rhizospheres under low-nutrient availability. (A) Recruitment plots showing hydroponic rhizosphere metagenomes six-frame translations and its amino acid identity against *Luteolibacter, Flavobacterium, Sphingopyxis*, and *Hyphomicrobium* pangenomes. Blue dots are forward and pink ones reverse matches. (B) Heatmap of nitrogen-fixation proteins showcasing the relative abundance in hydroponic versus soil tomato metagenomes. A green heatmap is also included to present a taxonomic revision of each protein, detailing the taxa associated with each nitrogen-fixation protein and their relative abundances. (C) Coverage and average amino acid identity graphs for *Methyloversatilis*. This panel highlights the genomic representation and sequence conservation of *Methyloversatilis* in the metagenomes, underscoring its significance in nitrogen metabolism within the hydroponic system.

We constructed genus-level pangenomes using the reference genomes of *Luteolibacter, Flavobacterium, Sphingopyxis*, and *Hyphomicrobium*, which were identified as key taxa in the tomato root under low-nutrient availability through 16S rRNA analysis. These pangenomes served as anchors to recruit metagenomic reads, verifying their presence in the hydroponic rhizosphere. Comparison with hydroponic metagenomic reads showed high similarity, with amino acid identities ranging from 70% to 100%, confirming their involvement in the hydroponic rhizosphere (Fig. 5A).

Additionally*, Luteolibacter* was the most prevalent genus in hydroponic taxonomy, with significant representation from *Luteolibacter luteus* (12.14%) and *Luteolibacter ambystomatis* (11.9%). Other notable classifications included bacteria (4.37%), Proteobacteria (1.61%), *Pseudomonas mexicana* (1.53%), and *Methyloversatilis* sp. RAC08 (0.93%) (Table S19 and Fig. S7).

We also identified nitrogen-fixation-related protein families using Level 4 SEED categories, showing an increased abundance in hydroponic treatments compared to soil metagenomes, especially the nitrogenase complex (Fig. 5B). Taxonomic identification with Kraken2 revealed that the *Methyloversatilis* genome contained all identified Nif-related proteins, followed by unclassified Cyanobacteria and *Rhizobacter* sp. AJA081-3. An analysis of the *Methyloversatilis* pangenome confirmed its presence in hydroponic metagenomes, with amino acid identity percentages ranging from 70% to 100% (Fig. 5C).

## Discussion

### Microbiome diversity and plant growth

As expected, the highest microbiome diversity was observed in treatments (Fig. 2C), while control groups f50, f100, and the NC group showed lower diversity due to the lack of additional microbiological input. However, the Shannon diversity values in our treatments (4.92 ± 0.29) were lower than those reported in previous studies of soil-grown tomatoes. Cai et al. (2017) reported an average diversity of 5.38, and Barajas et al. (2020) reported values between 6.21 and 7.75. In hydroponic conditions, Shannon values ranged from 3.2 to 4.3, as Anzalone et al. (2022) noted, suggesting that the confined environment of hydroponics limits microorganism movement toward plant roots, reducing diversity. Anzalone et al. (2022) also reported a decrease in fungi in hydroponics compared to soil-grown tomatoes.

The fertilized controls showed predictable growth patterns, with the f100 controls achieving the highest biomass, indicating their nutritional needs were fully met. However, they exhibited lower bacterial diversity than the f50 controls (Fig. 2C), supporting van der Heijden et al. (2008), who hypothesized that optimal nutrient conditions reduce the need for plants to recruit a diverse microbial community. Treatments with 50% fertilization and inoculation showed greater bacterial diversity and biomass than the f50 and NC controls. This suggests that plants recruit bacteria under nutrient-limited conditions to support their growth. Our findings indicate that reduced nutrient availability increases the recruitment of beneficial microbial diversity, enhancing plant productivity. Similar results have been reported in rice and soybean under low-nutrient conditions (Sinong et al. 2021, Wang et al. 2024) and in maize, where the root microbiome is linked to plant genetic variability under low nitrogen conditions (Meier et al. 2022).

The variation in bacterial communities between treatments and controls (Fig. 2E) demonstrates a link between the microbiome and plant phenotype, indicating that our system facilitates the recruitment of rhizosphere communities from the inoculum. Our study found a high proportion of the phyla Proteobacteria and Bacteroidota in both control and treatment groups (Fig. 2D), consistent with previous reports on tomatoes (Cai et al. 2017, Zolti et al. 2019). These phyla are typically dominant in the rhizospheres of tomatoes and other *Solanum* spp. (Zolti et al. 2019, Barajas et al. 2020).

Verrucomicrobiota was the third most abundant phylum in the tomato rhizosphere, consistent with findings from hydroponic tomato systems (Vargas et al. 2021). In rice studies, Verrucomicrobiota has been linked to root growth-promoting traits (Bünger et al. 2020). Our research showed enrichment of Verrucomicrobiota in treated groups compared to controls, suggesting that this phylum may play an important role in supporting plant development under nutrient scarcity.

*Luteolibacter*, a Verrucomicrobiota genus enriched in treatment groups, correlated positively with plant phenotypes and was part of the Hydroponic Core Microbiome (HTCM). Found in diverse environments like potato and leek rhizospheres and marine settings (da Rocha et al. 2010, Park et al. 2013), *Luteolibacter* has potential as a plant growth-promoting activity in heavy metal-contaminated environments (Zadel et al. 2020). Proteins related to stress responses, such as those protecting against reactive oxygen species (ROS), were identified in *Luteolibacter*. While *Flavobacterium* (Bacteroidota) was the most abundant genus across treatments and controls, it did not directly correlate with plant phenotypic traits (Tables S5 and S7), indicating a key microbiome role despite the lack of a direct relationship with plant phenotype, aligning with previous observations (Anzalone et al. 2022).

*Sphingopyxis* (Alphaproteobacteria) demonstrated the highest positive correlations with plant traits (Fig. 3A) and was overrepresented in treatments (Table S9). Known for producing indole acetic acid and enhancing plant growth, its association with QTLs in tomatoes underscores its significant role in root microbiome (Oyserman et al. 2022).

*Hyphomicrobium* (Alphaproteobacteria), although not predominant, showed significant correlations with plant traits (Table S7). Its role in the tomato rhizosphere is likely enhanced by its facultative methylotrophy, allowing it to thrive in hydroponic systems by utilizing methane (Martineau et al. 2013).

Genera exclusive to treatments, such as *Akkermansia, Methylocapsa, Arenibacter, Marimicrobium, Syntrophomonas, Nitrosococcus, Thiohalobacter*, and *Kaistia*, could be critical under tomato nutrient-poor conditions. *Kaistia*, which correlated positively with plant traits and was identified in the HTCM, is known for enhancing plant growth under phosphorus limitation (Liu et al. 2023). Metabolites from *Kaistia* have been reported to modulate the biofilm and motility of *Methylobacterium* (Usui et al. 2020a, b), which was also found in the HTCM and positively correlated with plant phenotype.

### Proteins in hydroponic tomatoes under a low-nutrient concentration

We identified 1 984 648 protein families with 70% identity in hydroponic treatments targeting plant growth promotion. Proteins overrepresented in hydroponic metagenomes spanned various COG categories, with “Replication and Repair of DNA (L)” being most prevalent (Fig. S4C). Bacterial communities likely use these proteins to repair DNA damage from ROS under low oxygen hydroponic conditions. Notable proteins included catalase, cytochrome C peroxidase, and Vanadium-dependent haloperoxidase, known for ROS protection. Chaperones ClpB and GroEL highlighted adaptation mechanisms crucial in oxygen-limited conditions and key to survival in hydroponics.

The HTCMe comprised 48 116 protein families in all conditions, while the TCMe included only 663 families. The most prevalent HTCMe protein, the IS110-like element ISPa71 family transposase, supports the hypothesis that the rhizosphere facilitates horizontal gene transfer (Maheshwari et al. 2017), allowing DNA rearrangements that help bacteria adapt to environmental changes (Lugtenberg and Dekkers 1999). The HTCMe core features proteins related to plant growth, such as enzymes involved in nitrogen fixation (Table S16).

HTCMe includes the BamA/TamA family outer membrane protein and TamB domain. TamA and TamB are part of the translocation and assembly module (TAM) subunits, which assemble outer membrane proteins related to adhesion and biofilm formation in bacteria (Josts et al. 2017). These proteins are crucial for infection and host colonization (Heinz et al. 2015), indicating TAM’s potential role in colonizing tomato roots.

Our study also explored the pangenomes of microbial genera in the hydroponic root metagenomes, confirming their presence. Genes within *Luteolibacter* and *Sphingopyxis* contigs included those related to multidrug resistance efflux pumps and chaperones, which may protect bacteria from plant-derived antibacterial compounds (Alvarez-Ortega et al. 2013, Paço et al. 2016). This finding suggests that these efflux pumps play a crucial role in the successful colonization and persistence of these bacteria in the hydroponic rhizosphere.

### Nitrogen fixation and assimilation in the bacterial community

We found that nitrogen-fixation genes are more abundant in our tomato hydroponic system than in soil-grown, emphasizing the role of microbial communities under nutrient deficiency conditions. For instance, nitrogenase-stabilizing protein NifW is crucial for maintaining nitrogenase activity in aerobic conditions, highlighting its significance for diazotrophic bacteria in oxygen-rich environments (Nonaka et al. 2019). Another protein enriched in hydroponics was the nitrogenase molybdenum–iron protein subunits, along with NifB, NifE, and NifN, which are involved in the biosynthesis of the nitrogenase cofactor (Rettberg et al. 2020). These findings suggest that nitrogen fixation is a crucial mechanism by which the hydroponic microbiome supports plant growth, highlighting the recruitment of such proteins in the root microbiome under low-nutrient availability.

Nitrite can be reduced to ammonia by ferredoxin-nitrite reductase (NirA) in *Luteolibacter* or by nitrite reductase NADPH large subunit (NasD) in *Luteolibacter, Sphingopyxis*, and *Methyloversatilis*. This involvement in the nitrogen cycle highlights the collaborative nature of microbial communities in facilitating plant nitrogen acquisition, which is essential for optimal growth. *Methyloversatilis* plays a unique role in the hydroponic system by potentially using methanol or methane for carbon and energy while contributing to nitrogen fixation (Doronina et al. 2014, Smalley et al. 2015). Methane in anoxic zones of the hydroponic rhizosphere may enable *Methyloversatilis* to perform functions such as nitrate reduction, illustrating the complex interplay of microbial activities that may contribute to supporting plant growth (Sun et al. 2016).

## Conclusions

This research highlights the importance of understanding root microbiome dynamics under nutrient deficiency as a pathway to reduce fertilizer use and enhance agricultural productivity and sustainability. Excessive fertilization can lead to nutrient pollution, greenhouse gas emissions, biodiversity loss, and health impacts. Our model shows reduced nutrient availability increases microbial diversity, enhancing plant productivity under controlled conditions. By leveraging the microbiome under low-nutrient availability for plants, we can enhance plant growth and resilience, making agricultural practices more sustainable and environmentally friendly.

## Supplementary Material

fiaf019_Supplemental_Files

## Data Availability

Datasets generated for this study can be found in NCBI Bioproject PRJNA984704. Amplicon 16S rRNA sequencing data is available from SRR24973180 to SRR24973211 accessions. Raw shotgun sequences are available from SRR24973791 to SRR24973793 accessions. The bioinformatic protocols are available on GitHub: genomica-fciencias-unam/tomato-hydroponics. Raw Data necessary for the analyses at FigShare: 10.6084/m9.figshare.26543761.
