# Supplementary figures and images for: Rhizosphere microbiome influence on tomato growth under low-nutrient settings
Source: FEMS Microbiol Ecol. 2025 Feb 25;101(3):fiaf019. doi: 10.1093/femsec/fiaf019 (PMC11879582; doi:10.1093/femsec/fiaf019)

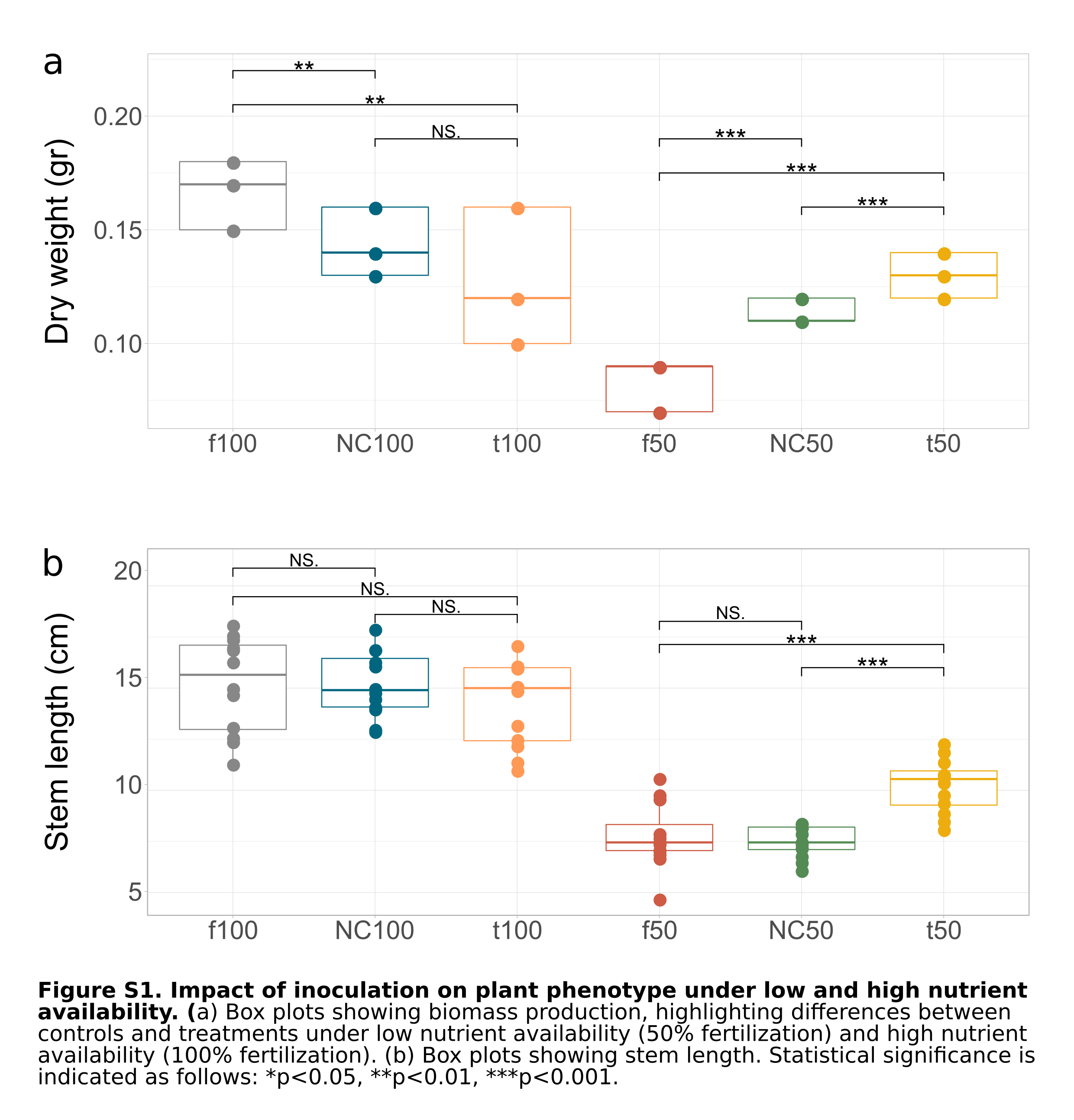

Supplement: fiaf019_Supplemental_Files [file fiaf019_supplemental_files.zip › Fig_S1.png]

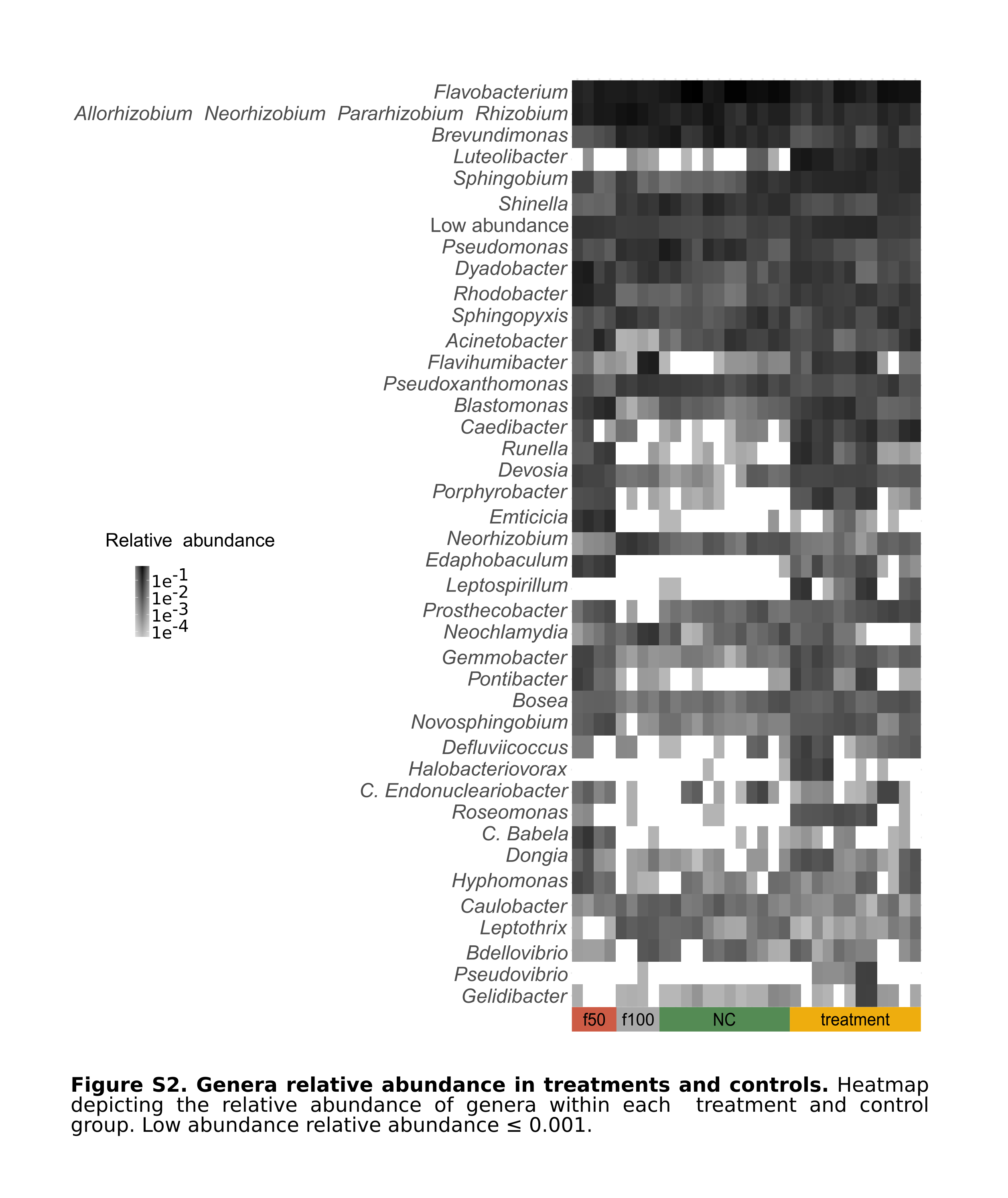

Supplement: fiaf019_Supplemental_Files [file fiaf019_supplemental_files.zip › Fig_S2.png]

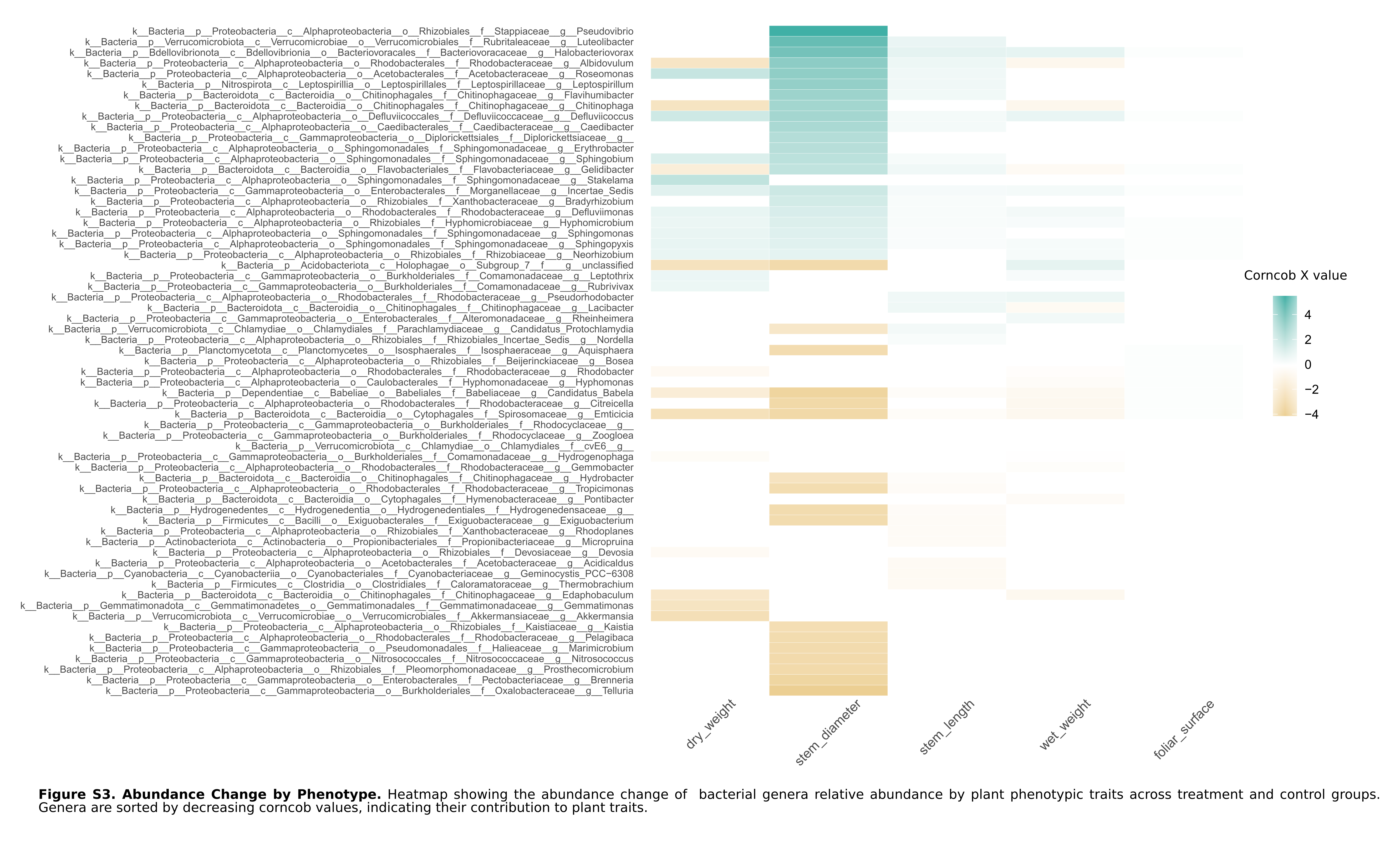

Supplement: fiaf019_Supplemental_Files [file fiaf019_supplemental_files.zip › Fig_S3.png]

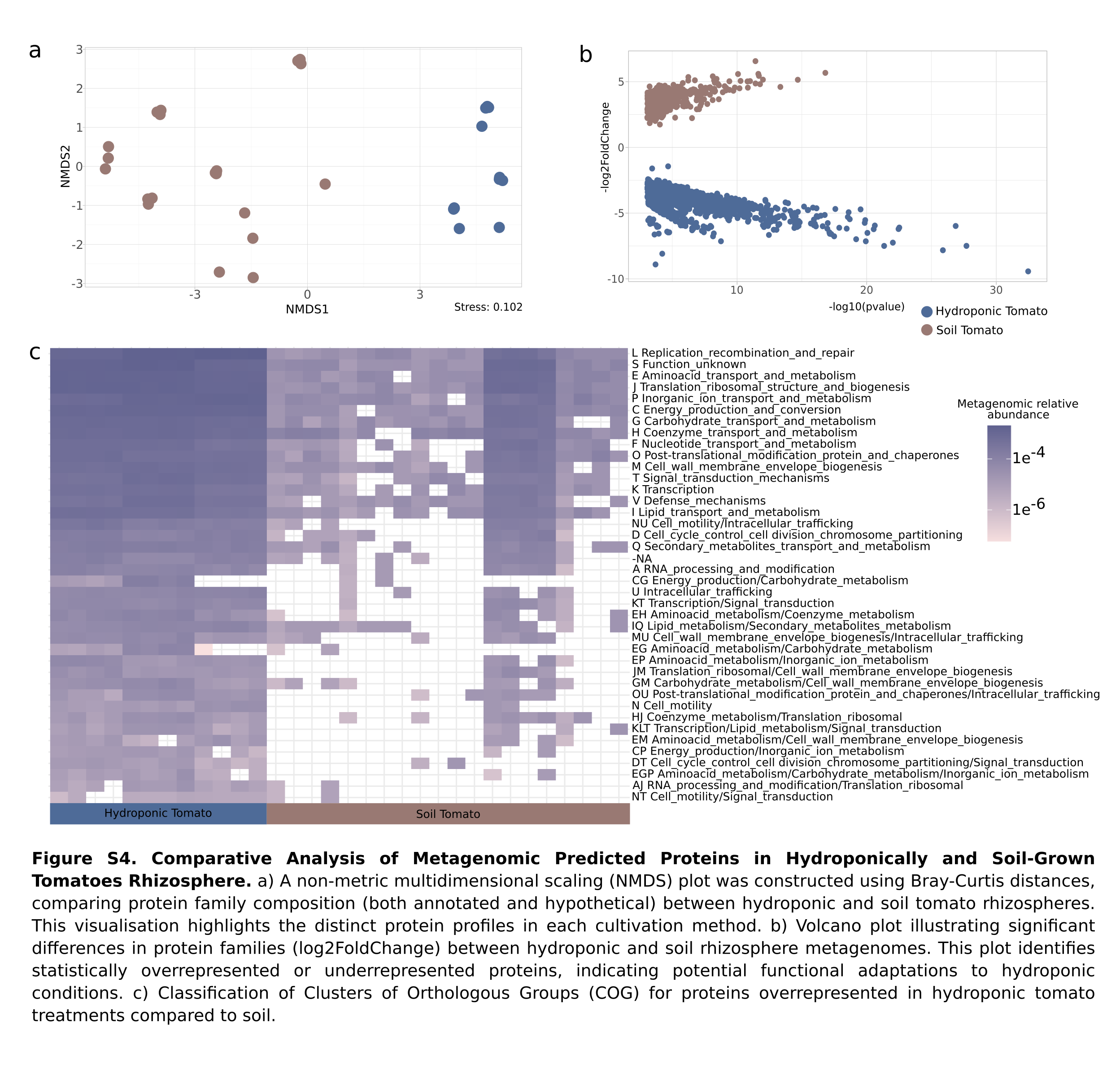

Supplement: fiaf019_Supplemental_Files [file fiaf019_supplemental_files.zip › Fig_S4.png]

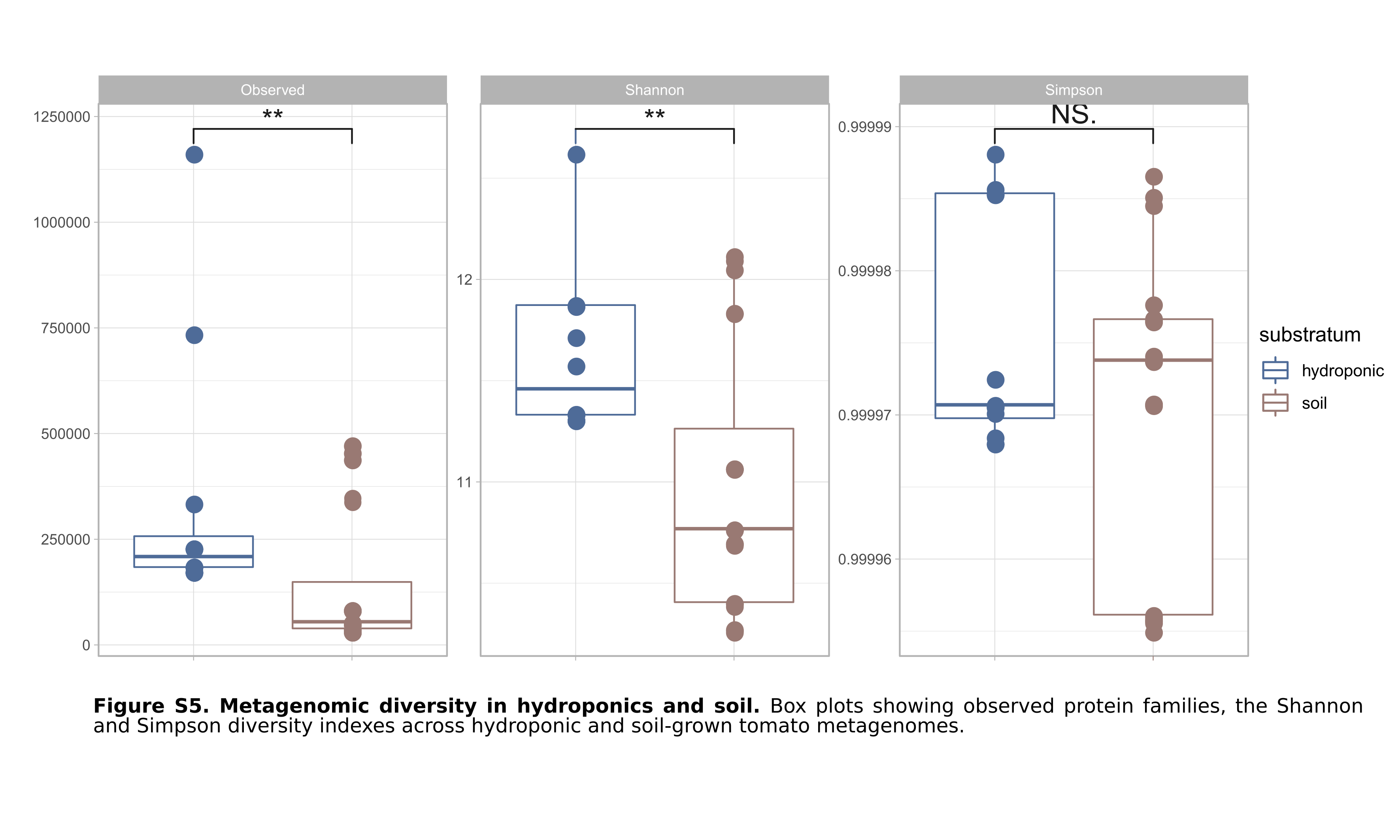

Supplement: fiaf019_Supplemental_Files [file fiaf019_supplemental_files.zip › Fig_S5.png]

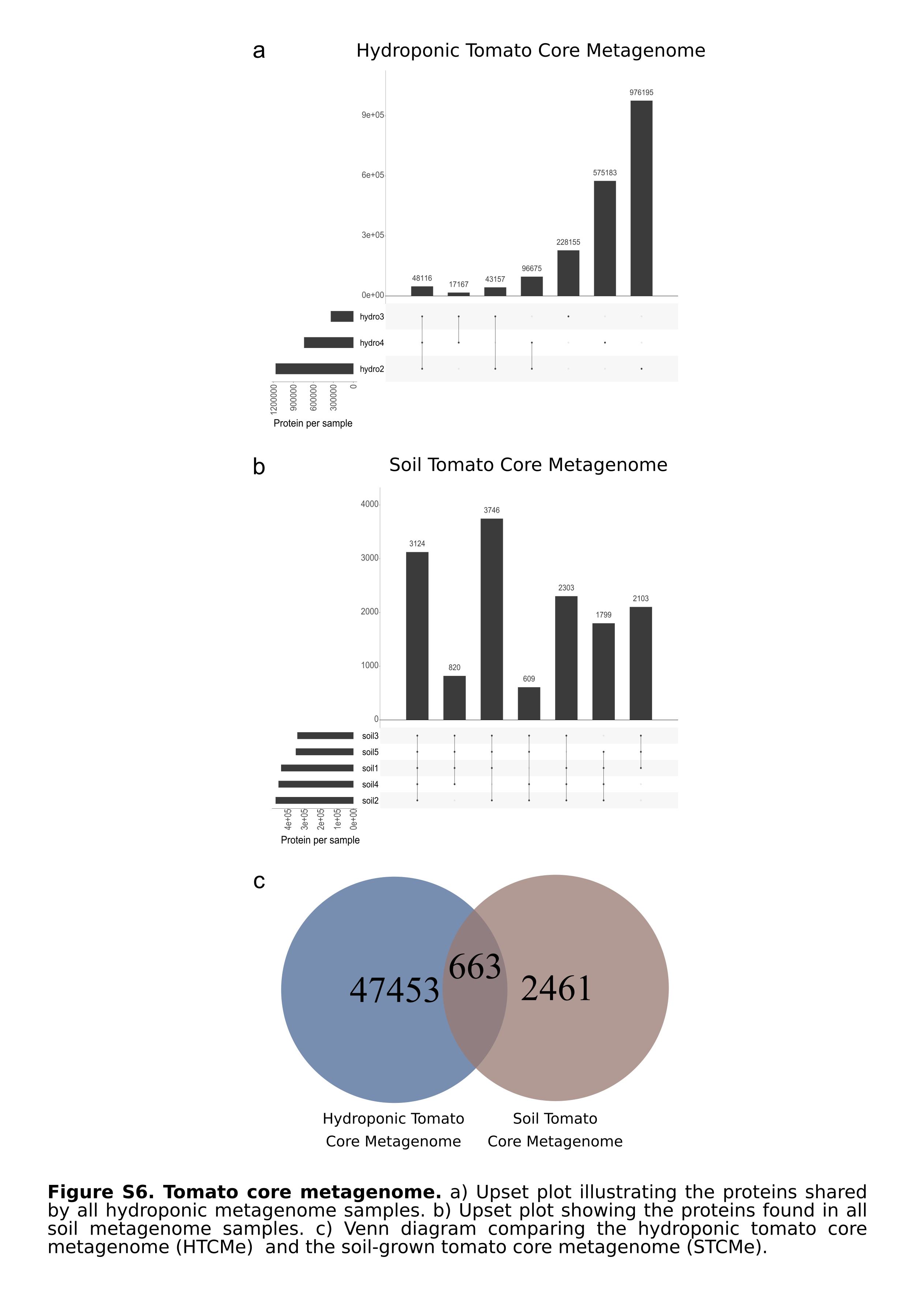

Supplement: fiaf019_Supplemental_Files [file fiaf019_supplemental_files.zip › Fig_S6.png]

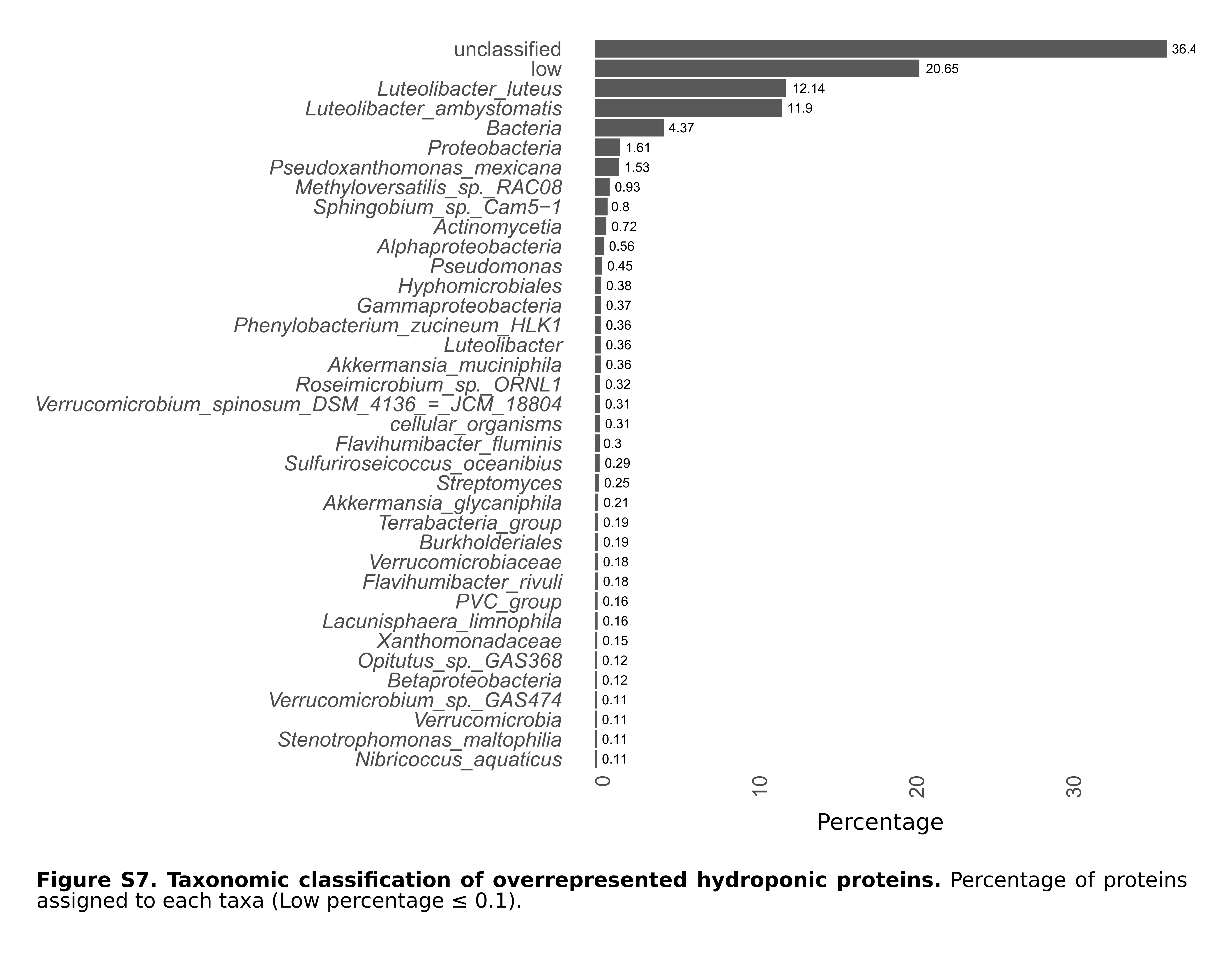

Supplement: fiaf019_Supplemental_Files [file fiaf019_supplemental_files.zip › Fig_S7.png]
